# Cross-sectional retrospective analysis of clinical characteristics of chronic hepatitis B patients with oral antiviral treatment in eastern China

**DOI:** 10.1186/s12985-021-01491-6

**Published:** 2021-01-13

**Authors:** Jueqing Gu, Guodong Yu, Xiaoli Zhang, Shanyan Zhang, Huan Cai, Chanyuan Ye, Yida Yang, Dezhou Li, Zhaowei Tong, Huajiang Shen, Huazhong Chen, Feng Ding, Xijie Lai, Junyan Liu, Meiling Xu, Weiti Wu

**Affiliations:** 1 State Key Laboratory for Diagnosis and Treatment of Infectious Diseases, National Clinical Research Center for Infectious Diseases, Collaborative Innovation Center for Diagnosis and Treatment of Infectious Diseases, Zhejiang University School of Medicine First Affiliated Hospital, Hangzhou, 310031 CN China; 2 Department of Infectious Diseases, Ningbo No 2 Hospital, Ningbo, China; 3 Department of Infectious Diseases, Huzhou Central Hospital, Huzhou, China; 4 Department of Infectious Diseases, Affiliated Hospital of Shaoxing University, Shaoxing, China; 5 Department of Infectious Diseases, WenZhou Medical College Affiliated Taizhou Hospital, Wenzhou, China

**Keywords:** Chronic hepatitis B, Antiviral treatment, Comorbidity, Aging

## Abstract

**Background:**

In China, more than 20 million patients with chronic hepatitis B need antiviral treatment. Side effects of antiviral treatment such as renal complications can be problematic, particularly in an aging population.

**Methods:**

The data were retrospectively extracted from the hospital medical charts of five centers in eastern China from January 1 to December 31, 2018.

**Results:**

A total of 8309 patients with CHB was enrolled in this study. The median age of the patients was 46 years. The prevalence of diabetes mellitus, hypertension, and hepatic cirrhosis was respectively 3.49%, 4.42%, and 23.72%. The prevalence of these comorbidities increased with age (P < 0.001). Of the patients with CHB, 5332 had complete renal function results. Among them, patients with an estimated glomerular filtration rate of < 60 mL/min/1.73m^2^ accounted for 4.14%, and those with proteinuria for 8.33%. According to the definition of chronic kidney disease, the proportion of patients with chronic kidney disease was 11.37%. The prevalence of chronic kidney disease increased with age (P < 0.001). In a multivariate analysis, age group [odds ratio (OR) = 2.387], diabetes mellitus (OR = 1.486), hypertension (OR = 2.557), hepatic cirrhosis (OR = 1.295), and a history of exposure to adefovir dipivoxil (OR = 1.644) were significantly associated with CKD (P < 0.05). Among patients with CKD, 17.66% (107/606) had a history of lamivudine exposure, and 34.65% (210/606) had a history of nucleotide analogue exposure

**Conclusion:**

The management of Chinese patients with CHB should take into consideration age, previous medication history, and renal impairment.

## Introduction

Hepatitis B virus (HBV) infection is a major public-health challenge worldwide. In particular, China has a high rate of morbidity due to HBV infection. The rate of positivity for the HBV surface antigen (HBsAg) was approximately 6.1% in the general population of China in 2016, but only 19% of these patients were diagnosed [1]. More than 80 million patients are infected with the hepatitis B virus, and 20 million patients with chronic hepatitis B (CHB) are in need of antiviral treatment, but only a small proportion of patients with CHB receive treatment [1–3].

In 1992, the rate of HBsAg carriage in China was 9.75% [4]. To control the spread of HBV, the government carried out a series of national immunization programs. Due to hepatitis B vaccination, the incidence of HBV infection in children and young people has declined significantly [2, 5]. In an observational study of HBV infection in China from 2004 to 2014, the rate of infection was significantly lower in people less than 24 years of age but significantly higher in those more than 55 years of age [6]. Carriers of hepatitis B virus are aging, particularly those infected before the introduction of hepatitis B vaccination. A recent multi-center cohort study of patients with CHB in the United States showed that elderly patients had a higher incidence of liver and non-liver complications [7]. However, to our knowledge, the data on this phenomenon in China are sparse.

CKD should be monitored because it stems from long-term renal function damage, which is both painful for the patient and imposes a considerable burden on society. At present, whether HBV infection increases the risk of chronic kidney disease is still controversial [8–10]. A single-center Chinese study reported that only 7.9% of patients with CHB had CKD, less than the prevalence in the adult Chinese population in a national cross-sectional survey [11]. Therefore, the incidence of CKD in patients with CHB warrants further investigation. And because hepatitis B cannot be cured, long-term or even lifelong treatment is required. Therefore, the common side effects of long-term treatment, such as kidney complications, need to be evaluated [12]. In some patients with CHB, prolonged use of nucleotide antivirals, including adefovir dipivoxil (ADV) and tenofovir diester fumarate (TDF), leads to renal dysfunction [13–15]. In a retrospective study of 687 patients with CHB treated with ADV, 10.5% exhibited a 20% decrease in the estimated glomerular filtration rate (eGFR) relative to the baseline [16]. A large-scale real-world cohort study also showed that patients with CHB treated with TDF had a slightly increased risk of CKD [17]. However, the data on the prevalence of early renal injury in patients with CHB treated with antiviral agents are sparse.

The study sites were in Zhejiang Province, eastern China. The overall prevalence of chronic HBV carriage in Zhejiang Province was 11.61% in 1992 and 8.79% in 2006, both higher than the national average [18]. Regarding ethnicity, the Han predominates both in Zhejiang Province and nationwide. HBV genotypes B and C are prevalent in China, whereas genotypes A and D are rare [19]. Therefore, data from Zhejiang Province could reflect the HBV infection status nationwide. The economic development and healthcare system of Zhejiang Province are relatively advanced, and the provincial reimbursement program covers the cost of antiviral treatment for hepatitis B. Also, physicians and patients are aware of the importance of HBV suppression. The majority of patients with CHB eligible for antiviral treatment according to the China HBV guidelines begin treatment at the time of diagnosis. We gathered the information of these patients and determined the optimal treatment strategy.

## Methods

### Study population

This was a cross-sectional, multi-center study at five centers in eastern China—the First Affiliated Hospital of Zhejiang University; Ningbo No. 2 Hospital; Huzhou Central Hospital; Shaoxing Municipal Hospital; and Taizhou Hospital Affiliated to Wenzhou Medical College. The study protocol was approved by the Ethics Committee of The First Affiliated Hospital, College of Medicine, Zhejiang University.

After reviewing patient medical charts, patients with CHB who met the following criteria were enrolled in the study: age ≥ 18 years; HBsAg positive for > 6 months; eligible for antiviral treatment according to the 2015 China HBV Guidelines; visited one of study hospitals from January 1 to December 30, 2018 and on nucleotide/nucleoside antivirals; no co-infection with hepatitis A virus, hepatitis C virus, hepatitis D virus, hepatitis E virus, or human immunodeficiency virus; no liver dysfunction of other causes; no concomitant liver or non-liver malignancy; and no pregnancy or lactation.

### Data collection and definitions

The following data were extracted from electronic medical charts: demographics (age and sex); medical history [diabetes mellitus (DM) and hypertension identified by International Statistical Classification of Diseases and Related Health Problems, 10th Revision (ICD-10) code]; antiviral treatment (current nucleotide/nucleoside regimen, oral antiviral treatment duration and prior exposure); liver-related complications (identified by ICD-10 code); and liver cirrhosis (determined by liver histology or clinical, radiologic, or endoscopic evidence of portal hypertension [nodular contour on imaging, thrombocytopenia, splenomegaly, presence of varices, or clinical hepatic decompensation], or in the notes of the responsible physician).

Laboratory parameters were collected from the digital medical charts, if available. The most recent result was selected if multiple tests had been conducted during the study period. The laboratory parameters evaluated were as follows: HBV virological or serological markers [HBsAg, hepatitis B e antigen (HBeAg), anti-HBV core (anti-HBc), and HBV DNA level]; hematological indicators [white blood cell (WBC) count, platelet (PLT) count; serum aminotransferase (ALT) level, and serum creatinine (Scr) level]; eGFR [estimated using the Chronic Kidney Disease Epidemiology Collaboration (CKD-EPI) equation as recommended by the 2012 KDIGO guidelines]; and urinary tests [dipstick urinalysis and quantitative markers of proteinuria (uric micro-albumin)].

### Data analysis

Descriptive statistics were reported as proportions (%) for categorical variables and as means ± standard deviations or medians with interquartile ranges in parentheses for continuous variables. Categorical variables were evaluated using the χ^2^-test or Mantel–Haenszel χ^2^-test. For continuous variables, one-way analysis of variance (ANOVA) was applied if a normal distribution was observed; otherwise, the Kruskal–Wallis H-test was used. A multivariate logistic regression analysis was conducted to identify predictors of CKD. All statistical tests were two-sided and a value of P < 0.05 was taken to indicate statistical significance. All analyses were performed using Statistical Package for the Social Sciences (SPSS) software, version 22.0 (IBM Corporation, Armonk, NY, USA).

## Results

### Patient characteristics

#### Basic information and laboratory parameters

From January to December 2018, a total of 8320 patients with CHB was consecutively enrolled in this study. After excluding 11 patients less than 18 years of age, the records of 8309 patients were analyzed. The median age of the patients with CHB was 46.00 (37.00–55.00) years, and the male-to-female ratio was 2.08:1. Regarding laboratory parameters, 76.96% of the patients with CHB had HBsAg levels of  >  250 IU/mL (because a highly sensitive quantitative assay of HBsAg is not available at some hospitals, only the proportion of patients with HBsAg levels > 250 IU/mL was calculated). A total of 3326 (40.03%) patients tested positive for HBeAg. In 6334 (76.23%) of the patients with CHB, serum HBV DNA levels were < 30 IU/mL—i.e., undetectable, and the median HBV DNA level of the remaining patients was 3.12 (2.28–4.78) log10 IU/mL. In addition, the median ALT level, WBC count, and PLT count were 24.00 (17.00–35.00) U/L, 5.30 (4.30–6.40) 10E9/L, and 168 (122–214) 10E9/L, respectively (Table 1).

**Table 1:** Characteristics of the CHB patients

Chataceristics	Total
Number	8309
Age (years)	46.00 (37.00–55.00)
≥ 60 years old, n(%)	1293 (15.56)
Male, n(%)	5609 (67.51)
HBsAg > 250 IU/mL, n(%)	6395 (76.96)
HBeAg positive, n(%)	3326 (40.03)
HBV DNA undetectable, n(%)	6334 (76.23)
ALT (U/L)	24.00 (17.00–35.00)
WBC (10E9/L)	5.30 (4.30–6.40)
PLT (10E9/L)	168 (122–214)

Categorical variables were presented as count (percentage) and continuous variables were presented as median (interquartile range, IQR) or mean ± standard deviation (SD)

*HBsAg* hepatitis B virus surface antigen, *HBeAg* hepatitis B e antigen, *HBV* hepatitis B virus, *ALT* alanine transaminase, *WBC* white blood cell, *PLT* platelet

#### Age and comorbidities

Based on the categories defined by the World Health Organization, we divided the patients into the following four groups according to their age: 18–44, 45–59, 60–74, and 75–89 years [20]. The majority of the patients with CHB were young or middle-aged, whereas 15.56% (n = 1293) were more than 60 years of age.

**Fig. 1:**
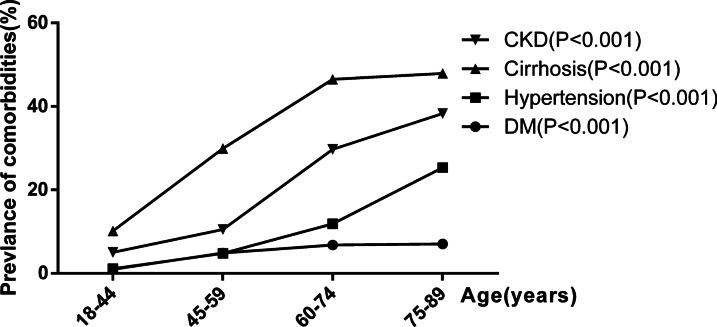
Comorbidities of patients with chronic hepatitis B. *CKD* chronic kidney disease, *DM* diabetes mellitus

The prevalence of diabetes mellitus, hypertension, and hepatic cirrhosis was respectively 3.49% (290/8309), 4.42% (367/8309), and 23.72% (1971/8309). The incidences of diabetes, hypertension, and cirrhosis in patients with CHB according to age group are shown in Fig. 1. The prevalence of diabetes (R = 0.123), hypertension (R = 0.191), and cirrhosis (R = 0.303) was linearly related to age (P < 0.01) and increased with age (P < 0.001).

### Antiviral treatment

#### Antiviral treatment status

The majority (71.73%) of the patients with CHB were taking entecavir (ETV), followed by TDF (16.22%). Among the remaining 12.05% of the patients, 2.68% were taking telbivudine (LDT), 2.15% were taking ADV combined with lamivudine (LAM), 2.14% were taking ADV, 1.31% were taking LAM, and 3.77% were taking other antiviral drugs. At the same time, a total of 22.16% of patients are taking treatment containing ADV or TDF.

#### Medium- or long-term antiviral treatment

Among the 8309 patients with CHB, the median duration of antiviral treatment was 24.00 (11.00–44.00) months. The antiviral status of patients treated for less and more than 24 months is listed in Table 2. For patients treated for > 24 months, the proportion of patients taking ETV was higher (75.76% vs. 67.93%; P < 0.001) and the proportion taking TDF was considerably lower (8.25% vs. 23.74%; P < 0.001) than for patients treated for < 24 months.

**Table 2:** Antiviral treatment status

Variable		Total (n = 8309)	Treatment duration ≤ 24 months (n = 4275)	Treatment duration > 24 months (n = 4034)	P value
Treatment duration (months)		24.00 (11.00–44.00)	11.00 (5.00–17.00)	46.00 (35.00–62.00)	–
ETV, n(%)		5960 (71.73)	2904 (67.93)	3056 (75.76)	< 0.001
TDF, n(%)		1348 (16.22)	1015 (23.74)	333 (8.25)	< 0.001
Others, n(%)		1001 (12.05)	356 (8.33)	645 (15.99)	< 0.001
LAM exposure, n(%)		1094 (13.17)	77 (1.80)	1017 (25.21)	< 0.001
Nucleotide analogue exposure n(%)		2556 (30.76)	1211 (28.33)	1345 (33.34)	< 0.001

*ETV* entecavir, *TDF* tenofovir disoproxil fumarate, *LAM* lamivudine

Among patients with CHB treated for > 24 months, the rate of ALT normalization (< 40 U/L in males and < 35 U/L in females) was 86.24%, and that of an undetectable serum HBV DNA level (< 30 IU/mL) was 89.11%. Among patients with CHB, the majority (75.76%) were taking ETV, whereas 25.21% had a history of exposure to LAM, 33.34% a history of exposure to nucleotide-based antivirals, and 35.92% had used two or more antivirals. A history of exposure to nucleotide-based antivirals was defined as having taken or currently taking TDF or ADV.

### Kidney function

#### General kidney function

Of the patients with CHB, 5332 had relatively complete renal function data. The median serum creatinine level and eGFR were 69.00 (59.00–80.00) μmol/L and 105.60 (92.64–114.57) mL/min/1.73m^2^, respectively. Of patients with relatively complete data, patients with an eGFR of < 60 mL/min/1.73m^2^ accounted for 4.14%, and patients with proteinuria for 8.33%. According to the definition of CKD, the proportion of patients with CKD was 11.37%. The prevalence of CKD increased with age (P < 0.001). Of the patients with CKD, 44.39% (269/606) had been treated for > 24 months, 17.66% (107/606) had a history of LAM exposure, 34.65% (210/606) had a history of exposure to nucleotide-based antivirals. Among them, 135 patients with hepatitis B had a history of ADV exposure and 87 patients had a history of TDF exposure. And the median duration of their antiviral treatment was 61.00(37–84) and 12.00 (6.00–24.25) months, respectively.

#### Risk factors for CKD

The relationship between CKD and related factors as deter mined by univariate and multivariate analyses is summarized in Table 3. Age group (18–44, 45–59, 60–74, and 75–89 years), HBsAg level (> 250 vs. ≤ 250 IU/mL), presence of HBeAg (positive vs. negative), diabetes mellitus, hypertension, hepatic cirrhosis, PLT count, and exposure to ADV were significantly associated with CKD (P < 0.001). ALT normalization rates were different between groups, but the difference was not statistically significant (P = 0.062). In a multivariate analysis, age group (odds ratio [OR] = 2.387, P < 0.001), diabetes mellitus (OR = 1.486, P = 0.025), hypertension (OR = 2.557, P < 0.001), hepatic cirrhosis (OR = 1.295, P = 0.008), and a history of exposure to ADV (OR = 1.644, P < 0.001) were significantly associated with CKD.

**Table 3:** Univariate and multivariate analysis of CKD

Variables	Univariate analysis	Multivariate analysis		
	P value	*B*	OR (95% CI)	P
Age groups	< 0.001*	0.870	2.387 (2.118–2.691)	< 0.001*
Gender	0.322	–	–	–
HBsAg > 250 IU–mL	< 0.001*	–	–	–
HBeAg positive	< 0.001*	–	–	–
HBV DNA undetectable	0.407	–	–	–
ALT normalization	0.062	–	–	–
Diabetes mellitus	< 0.001*	0.396	1.486 (1.052–2.099)	0.025*
Hypertension	< 0.001*	0.939	2.557(1.915–3.412)	< 0.001*
Hepatic cirrhosis	< 0.001*	0.259	1.295(1.070–1.567)	0.008*
WBC (10^9^/L)	0.439	–	–	–
PLT (10^9^/L)	< 0.001*	–	–	–
Treatment duration	0.304	–	–	–
ADV-experienced	< 0.001*	0.497	1.644(1.321–2.046)	< 0.001*
TDF-experienced	0.542	–	–	–

*OR* odds ratio, *HBsAg* hepatitis B virus surface antigen, *HBeAg* hepatitis B e antigen, *HBV* hepatitis B virus, *ALT* alanine transaminase, *WBC* white blood cell, *PLT* platelet, *ADV* adefovir dipivoxil, *TDF* tenofovir diester fumarate

*Statistical significance

#### Microalbuminuria

Of the patients with CHB, 651 were examined for urinary microalbumin, of which 19.35% (126/651) had microalbuminuria. The incidence of microalbuminuria increased with age (P < 0.001). The incidence of CKD was significantly higher in patients with CHB with microalbuminuria (P < 0.001), whereas 64.29% (81/126) of the patients with microalbuminuria had no comorbidity of CKD. Among the patients with CHB and microalbuminuria, 50.00% (63/126) had normal eGFRs (> 90 mL/min/1.73m^2^) and urinary protein levels. The patients with diabetes mellitus (P < 0.001), hypertension (P = 0.001), or cirrhosis (P < 0.001) had a higher incidence of microalbuminuria. In terms of antiviral status, patients with CHB with a history of exposure to TDF had a higher incidence of microalbuminuria (27.07.30% vs. 17.37%; P = 0.014), whereas a history of exposure to ADV (P = 0.0254), ALT normalization rate (P = 0.097), an undetectable HBV DNA level (P = 0.838), HBsAg level > 250 IU/mL (P = 0.307), and treatment for > 24 months (P = 0.0879) were not significantly related to microalbuminuria. In a multivariate analysis, CKD (OR = 5.212, P < 0.001), diabetes mellitus (OR = 2.908, P = 0.002), hepatic cirrhosis (OR = 2.089, P = 0.001), and a history of exposure to TDF (OR = 2.066, P = 0.004) were significantly associated with microalbuminuria.

## Discussion

This real-world study reflects the current status of patients with CHB in China. The median age of the patients with CHB was 46 years, and almost 16% were more than 60 years of age. Compared with two previous studies in China [5, 6] the patients with CHB were significantly older. Also, the incidence of hepatic cirrhosis and several non-liver complications increased with age. In patients older than 60 years of age, the incidence of complications, including hepatic cirrhosis, hypertension, diabetes, and CKD, was high. To some extent, this reflects the increasing age and incidence of complications of patients with CHB.

ETV was used by most of the patients. Because of its high viral suppression ability and low risk of resistance, the proportion of patients taking TDF has increased recently [21, 22]. Due to lower antiviral capacity, kidney and bone safety, drug resistance, and other factors, some antivirals have been relegated to second-line status [23, 24].

Many of the patients with CHB had a complicated medication history. Notably, some patients with CHB, particularly those treated for > 24 months, had achieved HBV suppression but > 25% had been exposed to LAM or nucleotide analogues. The history of previous medication might cause pre-existing mutations and lead to drug resistance [25–27]. And long-term use of ADV and TD might cause kidney damage [28].

Renal function is generally neglected during clinical monitoring. Renal function and urine tests were not performed for a considerable number of patients, and only ~ 8% underwent urinary microalbumin testing. In eastern China, routine 24-h urine protein quantitation or urine albumin-creatinine ratio (ACR) analysis is rarely performed, but urine dipstick testing is frequently conducted. Although there is no consensus on which test is superior to the rest [29, 30], because ACR results were lacking the result of the urine dipstick test was used to define proteinuria in this study. Approximately 11.4% of the patients with CHB were at risk of CKD, a larger proportion than in the general population [31]. Age, hypertension, hepatic cirrhosis, and a history of exposure to ADV were independent risk factors for CKD. Among these factors, hypertension had the highest OR and has a complex relationship with CKD. Indeed, hypertension may be both a cause and a result of CKD [32]. Age and diabetes mellitus were also important causes of CKD, consistent with prior studies [33]. Moreover, patients with CHB, hepatic cirrhosis, and a history of exposure to ADV were at increased risk of CKD. This may be related to hepatorenal syndrome caused by hepatic cirrhosis and the toxicity of some nucleotide antivirals to renal tubules, which leads to secondary osteoporosis and renal dysfunction [34, 35]. However, a history of exposure to TDF seemed to have no effect on renal function, which might be related to the exposure duration. Patients with a history of ADV exposure tended to have longer antiviral treatment time. In addition, the incidence of cirrhosis and rate of exposure to ADV were high among patients with CHB.

Due to the insufficient understanding of renal impairment, economic factors, and the inconvenience of urine retention, urinary microalbumin was tested in only a small proportion of the patients. Approximately 8% of CHB patients underwent urinary microprotein tests, which suggests that there is a huge bias in this study. Clinicians may be more inclined to perform urine microalbumin tests on patients who have already experienced renal impairment. Therefore, the factors that influence the incidence of microalbuminuria were not the same as the independent factors associated with CKD. However, half of the patients with microalbuminuria did not have abnormal urinary protein levels or eGFRs. This may be related to the high sensitivity of the urinary microalbumin test [36, 37].

This study had several limitations. The incidence of non-liver complications, including hypertension, diabetes, and CKD, was lower than in previous reports [38, 39], possibly due to biased selection of the patients with CHB and the lack of auxiliary examinations. The data were collected via medical chart review, and most of the participants were outpatients; thus, information on non-liver complications was frequently unavailable. In China, some patients with CHB return to community or local hospitals to continue their treatment; the relative inaccessibility of the medical records of other institutions resulted in incomplete data. The medications used are affected by the economic status of the patient, the time of drug listing, and the cost of the drug. As a cross-sectional study, it has some unavoidable limitations. We have adopted a multi-center, large sample approach to minimize bias. And no data for a normal population or for other years were available. This should be addressed in future studies.

## Conclusions

In conclusion, Chinese patients with CHB are aging (due to the high coverage of hepatitis B vaccination in China) and have an increasing incidence of complications. Therefore, the management of Chinese patients with CHB should take into consideration age, previous medication history, and renal impairment.

## Disclosure

The abstract of this paper was presented at the AASLD Conference as a poster with interim findings. The poster’s abstract was published in “Poster Abstracts” in Haptology Journal name “Cross-sectional Retrospective Analysis of Chronic Hepatitis B Patients in Eastern China” [ID 137173]: https://aasldpubs.onlinelibrary.wiley.com/doi/full/10.1002/hep.30941

## Data Availability

The datasets during and/or analysed during the current study available from the corresponding author on reasonable request.

## Abbreviations

HBV: Hepatitis B virus
HBsAg: Hepatitis B virus surface antigen
CHB: Chronic hepatitis B
HCC: Hepatocellular carcinoma
CKD: Chronic kidney disease
ADV: Adefovir dipivoxil
TDF: Tenofovir diester fumarate
eGFR: Estimated glomerular filtration rate
DM: Diabetes mellitus
ICD: International statistical classification of diseases
HBeAg: Hepatitis B e antigen
WBC: White blood cell
PLT: Platelet
ALT: Aminotransferase
Scr: Serum creatinine
ANOVA: One-way analysis of variance
SPSS: Statistical Package for the Social Sciences
ETV: Entecavir
LDT: Telbivudine
LAM: Lamivudine
ACR: Albumin-creatinine ratio

## References

[1] Razavi-Shearer D (2018). Global prevalence, treatment, and prevention of hepatitis B virus infection in 2016: a modelling study. Lancet Gastroenterol Hepatol.

[2] Liang X, Bi S, Yang W, Wang L, Cui G, Cui F, Zhang Y, Liu J, Gong X, Chen Y (2013). Reprint of: epidemiological serosurvey of hepatitis B in China—declining HBV prevalence due to hepatitis B vaccination. Vaccine.

[3] Yan YP, Su HX, Ji ZH, Shao ZJ, Pu ZS (2014). Epidemiology of hepatitis B virus infection in China: current status and challenges. J Clin Transl Hepatol.

[4] Xia GL, Liu CB, Cao HL, Bi SL, Zhan MY, Su CA (1996). Prevalence of hepatitis B and C virus infections in the general Chinese population. Results from a nationwide cross-sectional seroepidemiologic study of hepatitis A, B, C, D and E virus infections in China, 1992. Int Hepatol Commun.

[5] Liang X, Bi S, Yang W, Wang L, Cui G, Cui F, Zhang Y, Liu J, Gong X, Chen Y, Wang F, Zheng H, Wang F, Guo J, Jia Z, Ma J, Wang H, Luo H, Li L, Jin S, Hadler SC, Wang Y (2009). Epidemiological serosurvey of hepatitis B in China—declining HBV prevalence due to hepatitis B vaccination. Vaccine.

[6] Liu Z, Yang Q, Shi O, Ye W, Chen X, Zhang T (2018). The epidemiology of hepatitis B and hepatitis C infections in China from 2004 to 2014: an observational population-based study. J Viral Hepat.

[7] Liu A, Le A, Zhang J, Wong C, Wong C, Henry L, Nguyen MH (2018). Increasing co-morbidities in chronic hepatitis B patients: experience in primary care and referral practices during 2000–2015. Clin Transl Gastroenterol.

[8] Du Y, Zhang S, Hu M, Wang Q, Liu N, Shen H, Zhang Y, Yan D, Zhang M (2019). Association between hepatitis B virus infection and chronic kidney disease: a cross-sectional study from 3 million population aged 20 to 49 years in rural China. Medicine.

[9] Kim SE, Jang ES, Ki M, Gwak GY, Kim KA, Kim GA, Kim DY, Kim DJ, Kim MW, Kim YS (2018). Chronic hepatitis B infection is significantly associated with chronic kidney disease: a population-based, matched case-control study. J Korean Med Sci.

[10] Cai QC, Zhao SQ, Shi TD, Ren H (2016). Relationship between hepatitis B virus infection and chronic kidney disease in Asian populations: a meta-analysis. Ren Fail.

[11] Ning L, Lin W, Hu X, Fan R, Liang X, Wu Y, Shen S, Yu R, Sun J, Hou J (2017). Prevalence of chronic kidney disease in patients with chronic hepatitis B: a cross-sectional survey. J Viral Hepat.

[12] Wong GL, Seto WK, Wong VW, Yuen MF, Chan HL (2018). Review article: long-term safety of oral anti-viral treatment for chronic hepatitis B. Aliment Pharmacol Ther.

[13] Lee YS, Kim BK, Lee HJ, Dan J (2016). Pathologic femoral neck fracture due to fanconi syndrome induced by adefovir dipivoxil therapy for hepatitis B. Clin Orthop Surg..

[14] Wu C, Zhang H, Qian Y, Wang L, Gu X, Dai Z (2013). Hypophosphatemic osteomalacia and renal Fanconi syndrome induced by low-dose adefovir dipivoxil: a case report and literature review suggesting ethnic predisposition. J Clin Pharm Ther.

[15] Eguchi H, Tsuruta M, Tani J, Kuwahara R, Hiromatsu Y (2014). Hypophosphatemic osteomalacia due to drug-induced Fanconi's syndrome associated with adefovir dipivoxil treatment for hepatitis B. Intern Med.

[16] Kim YJ, Cho HC, Sinn DH, Gwak GY, Choi MS, Koh KC, Paik SW, Yoo BC, Lee JH (2012). Frequency and risk factors of renal impairment during long-term adefovir dipivoxil treatment in chronic hepatitis B patients. J Gastroenterol Hepatol.

[17] Wong GL, Chan HL, Tse YK, Yip TC, Lam KL, Lui GC, Szeto CC, Wong VW (2018). Chronic kidney disease progression in patients with chronic hepatitis B on tenofovir, entecavir, or no treatment. Aliment Pharmacol Ther.

[18] Yang SG, Wang B, Chen P, Yu CB, Deng M, Yao J, Zhu CX, Ren JJ, Wu W, Ju B (2012). Effectiveness of HBV vaccination in infants and prediction of HBV prevalence trend under new vaccination plan: findings of a large-scale investigation. PLoS ONE.

[19] Cui Y, Jia J (2013). Update on epidemiology of hepatitis B and C in China. J Gastroenterol Hepatol.

[20] World Health Organization. Global Burden of Disease 2000. WHO. https://www.who.int/healthinfo/paper50.pdf?ua=1. Data last accessed October 2002.

[21] Han Y, Zeng A, Liao H, Liu Y, Chen Y, Ding H (2017). The efficacy and safety comparison between tenofovir and entecavir in treatment of chronic hepatitis B and HBV related cirrhosis: a systematic review and meta-analysis. Int Immunopharmacol.

[22] Wang YJ, Yang L, Zuo JP (2016). Recent developments in antivirals against hepatitis B virus. Virus Res.

[23] Liu SH, Seto WK, Lai CL, Yuen MF (2016). Hepatitis B: treatment choice and monitoring for response and resistance. Exp Rev Gastroenterol Hepatol.

[24] Zoulim F, Durantel D, Deny P (2009). Management and prevention of drug resistance in chronic hepatitis B. Liver Int.

[25] Guo X, Wu J, Wei F, Ouyang Y, Li Q, Liu K, Wang Y, Zhang Y, Chen D (2018). Trends in hepatitis B virus resistance to nucleoside/nucleotide analogues in North China from 2009 to 2016: a retrospective study. Int J Antimicrob Agents.

[26] Feng B, Wei L, Chen M (2008). Dynamic changes of hepatitis B virus polymerase gene including YMDD motif in lamivudine-treated patients with chronic hepatitis B. Microbiol Res.

[27] Vutien P, Trinh HN, Garcia RT (2014). Mutations in HBV DNA polymerase associated with nucleos(t)ide resistance are rare in treatment-naive patients. Clin Gastroenterol Hepatol.

[28] Maggi P, Montinaro V, Leone A, Fasano M, Volpe A, Bellacosa C, Grattagliano V, Coladonato L, Lapadula G, Santantonio T (2015). Bone and kidney toxicity induced by nucleotide analogues in patients affected by HBV-related chronic hepatitis: a longitudinal study. J Antimicrob Chem.

[29] Hemmelgarn BR, Manns BJ, Lloyd A, James MT, Klarenbach S, Quinn RR, Wiebe N, Tonelli M (2010). Relation between kidney function, proteinuria, and adverse outcomes. JAMA.

[30] Park JI, Baek H, Kim BR, Jung HH (2017). Comparison of urine dipstick and albumin:creatinine ratio for chronic kidney disease screening: A population-based study. PLoS ONE.

[31] Zhang L, Wang F, Wang L, Wang W, Liu B, Liu J, Chen M, He Q, Liao Y, Yu X (2012). Prevalence of chronic kidney disease in China: a cross-sectional survey. Lancet (Lond, Engl).

[32] Hamrahian SM, Falkner B (2017). Hypertension in Chronic Kidney Disease. Adv Exp Med Biol.

[33] Romagnani P, Remuzzi G, Glassock R, Levin A, Jager KJ, Tonelli M, Massy Z, Wanner C, Anders HJ (2017). Chronic kidney disease. Nat Rev Dis Primers.

[34] Wei Z, He JW, Fu WZ, Zhang ZL (2016). Osteomalacia induced by long-term low-dose adefovir dipivoxil: Clinical characteristics and genetic predictors. Bone.

[35] Casado JL (2016). Renal and bone toxicity with the use of tenofovir: understanding at the end. AIDS Rev.

[36] Tsai JZ, Chen CJ, Settu K, Lin YF, Chen CL, Liu JT (2016). Screen-printed carbon electrode-based electrochemical immunosensor for rapid detection of microalbuminuria. Biosens Bioelectron.

[37] Chatzikyrkou C, Menne J, Izzo J (2017). Predictors for the development of microalbuminuria and interaction with renal function[J]. J Hypertens.

[38] Wong GL, Wong VW, Yuen BW, Tse YK, Luk HW, Yip TC, Hui VW, Liang LY, Lui GC, Chan HL (2019). An aging population of chronic hepatitis B with increasing co-morbidities—a territory-wide study from 2000 to 2017. Hepatology (Baltimore, MD).

[39] Nguyen MH, Lim JK, Burak Ozbay A, Fraysse J, Liou I, Meyer N, Dusheiko G, Gordon SC (2019). Advancing age and comorbidity in a us insured population-based cohort of patients with chronic hepatitis B. Hepatology (Baltimore, MD).

